# Making it “More Real”: Using Personal Narrative in Faculty Feedback to a Medical Student’s Reflective Writing – An Illustrative Exemplar

**DOI:** 10.15694/mep.2018.0000171.1

**Published:** 2018-08-14

**Authors:** Hedy S. Wald, Barrett Weiss

**Affiliations:** 1 Warren Alpert Medical School of Brown University/Boston Children's Hospital-Harvard Medical School; 2 Warren Alpert Medical School of Brown University

**Keywords:** interactive reflective writing, reflective practice, professional identity formation, medical humanities, professionalism, resilience

## Abstract

This article was migrated. The article was marked as recommended.

Reflective capacity is an essential characteristic of professionally competent clinical practice. Use of interactive reflective writing (IRW), ie. student writer/faculty feedback provider dyad and/or collaborative reflection in small group, to augment reflective practice instruction is well documented. IRW-enhanced reflection on experience contributes to meaning-making, leading to transformative learning within professional identity formation. Written formative feedback to trainees’ reflective writings can include personal anecdotes from faculty to enrich the educational value of feedback, used judiciously and subjected to a “filtering” process. We provide an exemplar of a third year Family Medicine clerkship student’s reflective writing about a clinical care experience that “mattered” as well as the faculty written feedback which included sharing a personal narrative resonating with themes emerging from the student’s reflective writing. Dual, mutually reinforcing identities/roles of medical educator and family cancer caregiver as educator emerged within the feedback.We include some post-IRW exercise student and educator reflections on the experience and impact of such sharing within an educational context. In this example, faculty drawing from both personal and professional experience to craft feedback supporting the becoming of a physician was experienced by the student writer as making the feedback more “real” and engaging, leading to perceived enhanced value of the educational exercise.

## Introduction — Interactive Reflective Writing in Medical Education

Interactive reflective writing (IRW), a component of medical humanities in medical education, can foster reflective capacity ( Wald and Reis, 2010, Sandars, 2009), a key aspect of professional competency ( Epstein and Hundert 2002). The use of reflection can foster empathic engagement ( Chen and Forbes, 2014) and humanistic practice and help guide health care practitioners as they encounter complexity inherent to clinical practice. IRW pedagogy elicits beliefs about professional responsibilities toward the underserved ( Ross et al. 2010), promotes cultural humility (Lie et al. 2011) and enhances understanding of ethical dilemmas ( Moon et al. 2013). As such, IRW-enhanced reflection on experience (both one’s own and that of patients) contributes to meaning-making, leading to transformative learning ( Mezirow, 1991) within professional identity formation ( Wald, 2015), an active, dynamic, and integrative developmental process involving establishment of core values, moral principles, and self-awareness (Holden et al. 2012).

The value of curriculum inclusion of IRW-guided critical reflection about complex experiences during medical training that may evoke challenging emotions has been emphasized as a means of creating safe pedagogical spaces for reflection ( Clandinin and Cave, 2008). Formal analytic frameworks have been developed and are now used internationally, ie. the “BEGAN” ( Reis et al. 2010, Wald et al. 2010) and the “REFLECT” rubric (Wald et al. 2012) to formatively assess reflective writing and guide faculty with crafting quality written feedback to trainees’ narratives to enhance depth and breadth of reflection ( Hall et al. 2012, Vanderbilt University 2014). The “interactive” nature of IRW-enhanced reflection includes writing to a structured writing prompt, facilitation of small group collaborative peer reflection on narratives that trainees are invited to share, and/or individualized written feedback from faculty to support grappling with the lived reality of medicine. Such “grappling with complexity” within a supportive community of practice ( Wenger, 1998) and skilled mentorship ( Wald 2015) for promoting learning has been described as a “positive hidden curriculum” ( Wald et al. 2018) given its potential for cultivating resilience/wellbeing and supporting healthy professional identity formation ( Wald, 2015). IRW has been described as a metaphorical “resiliency workout” with intellectual stretching, building emotional muscle, and fostering ethical fitness ( Murphy, 2012) for the “marathon” of clinical practice ( Wald et al. 2015). IRW within the medical humanities can support and challenge learners with a process of “questioning rather than providing answers, and problematizing the taken-for-granted,”( Bleakley 2015 p. 37) and can potentially enhance “joy in work” with heightening awareness of triumphs and accomplishments ( Wald et al. 2015). The development of critical reflective skills can reduce stress and foster wellness in medical students ( Lutz et al. 2013, Peterkin et al. 2012). Benefits of IRW curriculum for both trainees (Borgstrom et al. 2016, Wald et al. 2009) and faculty have been reported ( Wald 2008, Sukhato et al. 2016). Our implementation of an IRW curriculum within our Family Medicine Clerkship has been described ( Wald et al. 2015).

Written formative feedback to trainees’ RWs can include personal anecdotes to enrich the educational value of feedback, used judiciously and subjected to a “filtering” process ( Reis et al. 2010). Recently, a faculty small group facilitator and director of the Family Medicine Clerkship reflective writing curriculum (author HSW) reflected on her sharing of personal experience resonating with a third year medical student’s reflective writing when she provided written individualized feedback. She (author HSW) subsequently elicited “feedback on feedback” from this Clerkship trainee (author BW). Here we share author BW’s reflective narrative to a writing prompt within the IRW curriculum, author HSW’s written feedback to the narrative, and some post-IRW exercise trainee and educator reflections on the experience and impact of such sharing within an educational context.

## Illustrative Exemplar

### Reflective Writing Prompt

Write about a clinical care experience within this clerkship that mattered to you, that struck you. Describe the experience and reflect on its impact on your thoughts and feelings (personal and/or professional impact). You may consider writing about encountering dilemma, transformative learning experiences, eg. change in perspective, gaining emotional insight (if relevant), and/or confirmatory learning.

### Student Narrative

On my surgical rotation I was on surgery 3 team, which dealt primarily with oncology. By the time my family medicine rotation came around I had seen a large amount of cancer patients and the procedures and treatments they underwent. It started to become familiar, almost too familiar. Cancer became less of a scary word and more of just a condition to be treated. It started to lose the weight of the feeling of dread that accompanied the word. The surgery 3 service had a way of desensitizing me to a word that most people were very sensitive around. It wasn’t like I had forgotten that to most people the word was synonymous with death sentence or long chemo battle, I just felt more laissez-faire about it.

During my first weeks on family medicine I would not encounter any patients with recent or current cancer. A few patients here and there with a history of cancer, but not many. One day my preceptor would let me know in the morning that there was a patient coming in later who was young, and had recently gone into remission for Hodgkin’s lymphoma. I remember when my preceptor said the diagnosis it didn’t even strike me as odd, or scary, just something that was.

My preceptor and I decided to go in together to see the patient. We started off with some greeting and brief small talk before going into the interview as we always do. The patient looked young and full of energy, but almost seemed as if he was tired at the same time. I realized that for him, even though he was in remission, cancer was still a scary word. He had “beaten” it, but that only seemed to make him more nervous about it. As we started talking I noticed he would say “thank god no” after every negative review of systems. You could tell even though he had physically recovered, it would take him a while before he really mentally and emotionally recovered. And I started to realize that cancer wasn’t just a scary word because of the physical entity it is, but because of the fact that lots of people know the battle people go through when they have it. It is scary because for most people cancer is cancer, they don’t realize that thyroid cancer has a vastly different prognosis than gastric cancer. To them, they may know or have known someone with cancer and seen their battle. To them, cancer isn’t just a physical thing, it is a fight. It is an emotional battle.

I started to realize that I need to discover this desensitization earlier. Maybe it was helpful in my surgical rotation, maybe it wasn’t. Does it make me less empathetic? Does it make me more objective? Do I need to find a middle ground between the two. I feel like for me, the best option is in the middle. I can’t feel the same fear patients feel when hearing the word cancer, but I also need to not just remember, but really truly understand that patients see the word cancer as an emotional battle. It was the first time that I realized that knowing something isn’t just enough. Sometimes awareness of the fact that patients feel a certain way isn’t just enough. I have to be vigilant about continuing to understand that feeling. To be able to connect with that feeling.

I feel as if my experience with this patient was very eye opening, and that, hopefully, it will make me better able to discover when I have fallen into this trap again. I hope that now I will be able to always be in a happy middle ground.

### Faculty Feedback

Dear Barrett-

Thank you for your emotional candor. To your credit, I don’t think you fell into any “trap” as you are critically reflecting on your experience for transformative learning and through this process, developing mindful awareness going forward. It’s called Education.

Your narrative touches on a key dynamic within medical education and practice - how do we intentionally and empathically engage with sensitivity yet appropriately “desensitize” and distance for our professional demeanor and even self-preservation? How best to retain emotional “fuel” for the other patients we must see and be ready to engage with? In essence, how do we calibrate our emotional response? Where are you at with this at the current time?

Your development as a reflective practitioner, core to professional competency, comes through clearly in your narrative.. and your evident caring and compassion, as you wrote *“I started to realize that I need to discover this desensitization earlier. Maybe it was helpful in my surgical rotation, maybe it wasn’t. Does it make me less empathetic? Does it make me more objective? Do I need to find a middle ground between the two?”* David Leach’s article on “living the questions” within professional development (attached if you’d like to take a look) is one of my favorites and I thought of it reading your reflection-inviting questions. We may not have all the answers (at least right now within your professional identity formation) but asking the pertinent questions may help guide you along the trajectory.. and hopefully with some formative input from positive role models.

What may contribute to trainees’ and even seasoned practitioners’ emotional distancing? Might we feel somewhat vulnerable when not only witnessing suffering but being immersed in it? When we may need to find some comfort in healing vs curing? As you wisely allude to, cancer can challenge not only patients and their families in this way but also their caring practitioners. What strategies do you have for processing strong emotions, developing emotional resilience, and reducing stress through wellness activities?

On a personal note, I was very touched by your narrative as I am a family cancer caregiver. Indeed the “C” word is often if not always filled with terror and dread. I appreciate how seriously you took your experiences within oncology and your emotional reflection and exploration. Suffering patients and families will depend on you to attend to the physical and emotional components of this dreadful disease as well as treatment effects - this will be a learning and professional growth curve for you. While there is thankfully increased cancer survivorship, survivors (and patients in ongoing treatment) often have physical, emotional and financial needs that can continue.. we thus need primary care practitioners (along with oncologists) educated not only in treatment but in meeting needs of survivorship. I have published about my experience of my neurologist husband’s diagnosis of malignant brain cancer (eg. https://www.kevinmd.com/blog/2016/07/poised-abyss-wife-faces-neurologist-husbands-brain-cancer.html) and presented on caregiver needs for the National Academy of Medicine (online if of interest). We bring personal and professional stories to the work we do.

You wrote: *“It was the first time that I realized that knowing something isn’t just enough. Sometimes awareness of the fact that patients feel a certain way isn’t just enough. I have to be vigilant about continuing to understand that feeling. To be able to connect with that feeling.”* You hit it out of the park with this Barrett. So beautifully expressed. Thank you sincerely. And thank you for your reflective narrative. I wish you the best in your medical education journey and beyond,

Hedy Wald

## Further Reflections by Faculty and Student

**HSW**: I appreciated BW’s emotional candor within his reflective writing. I was struck by what his narrative evoked in me as well as the process of choosing to bring my personal experience, albeit a challenging one, into the learning and calibrating this. I hoped that this inclusion within the “feedback product” ( Wald et al. 2010) would enhance its educational value for BW. Reciprocally, I’ve written about realizing personal and professional growth as a medical educator within the IRW paradigm ( Wald 2008, Wald 2010), an example of relationship-centered education ( Rabow et al. 2014). This was supported more recently with teachers reporting that the activity of facilitating reflective learning through a RW exercise for medical students enhanced personal and professional fulfillment and renewed their enthusiasm ( Sukhato et al. 2016). While I shared as a medical educator within an educational context, I proceeded with caution given caveats about boundaries and physician disclosure within the clinical context ( Morse et al. 2008). Such sharing has, however, also been described as a means of fostering trust and rapport in the patient-physician relationship ( Candib 1987) and I would hope within the teacher-student relationship as well. While the sharing of my own challenging personal experience within the authenticity of what emerged for me within the reading (followed by a “filtering” process, Reis et al 2010) resulted in a somewhat longer than usual feedback “product,” I hoped it would help support BW’s reflective skills and even transformative learning ( Mezirow 1991). In addition to supplemental literature related to students’ reflective writing themes (including published reflective narratives, poetry, and/or peer-reviewed research) which I frequently attach, I also, in this instance, offered a link to my published first-person narrative about my cancer caregiver experience (Wald 2017). “Messages,” according to Prober and Heath (2012), “become stickier when they come in the form of a story that elicits emotion in readers or listeners.” Dual, mutually reinforcing identities/roles of medical educator and family cancer caregiver as educator ( Coulby and Jha 2015) thus emerged within the feedback.I invited BW’s “feedback on feedback” as well as feedback on the exercise in general. I was also curious whether he had read my published narrative as well as the article about professionalism and “living the questions” ( Leach 2014) which I had attached.

**BW**: The IRW exercise was valuable to create reflective space since students could potentially say “I’ll think about it later” when dealing with an experience such as the one I wrote about and then potentially not reap the benefits. Medical students are very busy, and even when they find free time, they often feel that there are additional tasks they *should* be doing. Reflection is one of those things that can often be an afterthought, something you “will” do at some point. Students *know* at some levelthat reflecting is important, but they often don’t *understand* what reflection will and should do for them or don’t quite connect with it. My reflecting on potential for “desensitization” was useful for self-awareness and recognizing the value of an ongoing process of “self-surveillance” ( White et al. 2006) within patient care. Designated time devoted to reflection offers an opportunity for students to engage with their experiences and feelings.

I appreciated being given the option to read the link about Dr. Wald’s (author HSW) experience as well as Dr. Leach’s article. Even the option to continue my self-reflection was empowering and oddly stress relieving. The written feedback motivated me to dive further into the additional publications provided and was a stimulus for continued reflection on other perspectives. In essence, my growth had not ended with my first write up.

Dr. Wald’s (author HSW) weaving of personal story into the professional narrative within the feedback made it more real and was refreshing. With this authenticity and level of sincerity, I felt I wasn’t just engaging in an intellectual exercise but also an emotional one. Her feedback to my perspective as a medical student validated the ideas I wrote about and furthered my understanding of a cancer caregiver’s experience. Her personal story was unexpected but appreciated. It spurred my thinking both about her experience as well as more broadly, about my clinical work with patients - what had I done “right” and what could I have done differently or improved. Reflection has become a part of my career now, not just something I do because I know I should.

## Conclusion

The power of the pen.. and the opportunity for a medical educator to draw from both personal and professional experience to support the becoming of a physician can be gratifying and even “reciprocally illuminating” ( Kneebone, 2015). For the trainee, when relevant, it may even make the feedback process “more real” to support one’s development as a reflective, resilient, and humanistic healthcare professional.

## Take Home Messages

• Use of interactive reflective writing (RW) to foster reflective capacity supporting professional identity formation in medical education is well documented.
• Reflective capacity is an essential characteristic of professionally competent clinical practice.
• Written formative feedback to trainees’ RWs can include personal anecdotes from faculty to enrich the educational value of feedback, used with a “filtering” process.
• Feedback to students’ RWs that is informed by insights derived from faculty dual roles/identities, eg. as medical educator and a family cancer caregiver, can be experienced by a student as more “real” and engaging.
• “Feedback on feedback” can illuminate students’ personal and professional growth.

## Notes On Contributors

Hedy S. Wald, PhD, is a Clinical Professor of Family Medicine, Warren Alpert Medical School of Brown University; Director, Residency Resilience/Wellbeing, Residency Programs in Child Neurology & Neurodevelopmental Disabilities, Boston Children’s Hospital - Harvard Medical School. She conducts workshops internationally on interactive reflective writing-enhanced reflection as well as on promoting resilience/wellbeing.

Barrett Weiss, BA is a fourth-year student at Warren Alpert Medical School of Brown University. He completed undergraduate studies with a neuroscience concentration at Brown University. He fenced competitively for Brown University and continues to fence during his medical school training. He plans to pursue a career in Physical Medicine and Rehabilitation.
