# Supplementary material for: Making it “More Real”: Using Personal Narrative in Faculty Feedback to a Medical Student’s Reflective Writing – An Illustrative Exemplar
Source: MedEdPublish (2016). 2018 Aug 14;7:171. [Version 1] doi: 10.15694/mep.2018.0000171.1 (PMC10701800; doi:10.15694/mep.2018.0000171.1)
Supplement: Peer review history

# Reviewer report: Reviewer response for version 1

**DOI:** 10.21956/mep.19631.r29222

**Published:** 2018-09-13

**Authors:** Ian Walsh

**Affiliations:** 1 Queen's University Belfast

This review has been migrated. The reviewer awarded 5 stars out of 5 I have to admit that, at first blush, I presumed this was to be a description of reflective learning in written format. I was greatly mistaken, as this represents a seismic shift far from and deeper to, an area that many of us pay lip service to. The paper's findings are particularly timely in the portfolio era. The authors describe deep learning and rich interaction and what becomes strikingly apparent are the dynamics and dynamism of the interaction. It is apparent that IRW fosters a pastoral and developmental approach to learning. Is the interaction more effective/relevant when between similarly matched participants, or does the dynamic of experienced educator with relatively inexperienced learner provide for a more fertile exchange? Following on from that, does IRW lend itself better to the postgraduate arena?

Reviewer Expertise:

NA

No decision status is available

# Reviewer report: Reviewer response for version 1

**DOI:** 10.21956/mep.19631.r29223

**Published:** 2018-08-14

**Authors:** Trevor Gibbs

**Affiliations:** 1 AMEE

This review has been migrated. The reviewer awarded 5 stars out of 5 We talk about feedback and reflection constantly within our curricula discussions, but I agree with what I feel that this paper bestows on our activity- we tend to use the words without thinking what they mean and how its action is structured.I enjoyed reading this personal view very much and learned a lot. As I discuss feedback extensively with the students I have the pleasure to teach, I try not to miss out the concept of feed-forward- what will happen as a result of the feedback. My feed-forward activities are often of a practical nature, an action, a new learning from reading, the development of a skill. As the author so wisely points out, this need not always be the case- it can be a thought process, a real inward reflection that can be manifested within the "thinking" of the student. Yes, maybe an action may follow, but that internalisation is so important a step, and maybe one that we omit or discard in exchange for a clear action.The second thought that I have now in reading this paper is how- how do we train faculty to be able to so carefully give that feedback to the student, how do faculty develop such such a talent as the author clearly shows.I doubt it can be taught in a stand alone course, I doubt if it exists without some sort of catalyst in the teachers / faculty member's past experience.And my final thought, why are are reticent about opening up to our students, sharing our life experiences with the student, exposing our real caring self.I sometimes feel we are afraid to let down those barriers , afraid to expose ourselves for what we are , what we feel, what we care about. Does medicine as we know it knock this out of us or is it the system we work in.A very well placed paper that I hope causes many readers to reflect upon themselves and perhaps their styles of teaching. A must-read for all involved in healthcare education

Reviewer Expertise:

NA

No decision status is available

# Reviewer report: Reviewer response for version 1

**DOI:** 10.21956/mep.19631.r29225

**Published:** 2018-08-14

**Authors:** Ronald M Harden

**Affiliations:** 1 AMEE

This review has been migrated. The reviewer awarded 4 stars out of 5 I did not find this paper an easy read but it is certainly worth the effort. A definition of what the authors describe as interactive reflective writing at the beginning of the article would have been helpful. For me the key and important messages were the value of reflective writing, the importance of feedback and the benefits of personalised feedback. It is very useful to see an example of the writing, the feedback and the response to the feedback. All concerned are to be congratulated. The feedback provider obviously has special skills and a personal story relating to the topic. While there are important messages I am not sure how generalisable is the approach described. How many teachers could have provided the feedback as described and what training is required? Perhaps this could be the subject of a further study. In the meantime we could try to follow this powerful example.

Reviewer Expertise:

NA

No decision status is available

# Reviewer report: Reviewer response for version 1

**DOI:** 10.21956/mep.19631.r29224

**Published:** 2018-08-14

**Authors:** Dujeepa D. Samarasekera

**Affiliations:** 1 National University of Singapore

This review has been migrated. The reviewer awarded 4 stars out of 5 Interesting paper. At times difficult to comprehend. However, enjoyed reading the paper which highlights the importance of feedback, having a clear understanding of what and how to do it effectively and new approaches for the faculty to adopt. It also highlights the value of faculty development to engage our students effectively. It would be very interesting to see how many of our faculty would use this approach esp opening up with one's own personal examples. Good article for educators.

Reviewer Expertise:

NA

No decision status is available
